# Relationships of Telomere Homeostasis with Oxidative Stress and Cardiac Dysfunction in Human Ischaemic Hearts

**DOI:** 10.3390/antiox10111750

**Published:** 2021-11-01

**Authors:** Estefanía Tarazón, Lorena Pérez-Carrillo, Isaac Giménez-Escamilla, Pablo Ramos-Castellanos, Luis Martínez-Dolz, Manuel Portolés, Esther Roselló-Lletí

**Affiliations:** 1 Myocardial Dysfunction and Cardiac Transplantation Unit, Health Research Institute Hospital La Fe (IIS La Fe), 46026 Valencia, Spain; lorena_perezc@iislafe.es (L.P.-C.); igies@alumni.uv.es (I.G.-E.); pramosca@navarra.es (P.R.-C.); martinez_luidol@gva.es (L.M.-D.); portoles_man@gva.es (M.P.); 2 CIBERCV, Institute of Health Carlos III, C/Monforte de Lemos 3–5, Pabellón 11, Planta 0, 28029 Madrid, Spain; 3 Heart Failure and Transplantation Unit, Cardiology Department, University and Polytechnic La Fe Hospital, 46026 Valencia, Spain

**Keywords:** ischaemic cardiomyopathy, telomere, *TERRA* and *GUARDIN*, oxidative stress response

## Abstract

Although the roles of telomeres and oxidative stress in ischaemic cardiomyopathy (ICM) are known, mechanisms of telomere homeostasis and their relationship with oxidative stress are incompletely understood. We performed two RNA-seq analyses (mRNA *n* = 23; ncRNA *n* = 30) and protein validation on left ventricles of explanted hearts from ICM and control subjects. We observed dysregulation of the shelterin and cohesin complexes, which was related to an increase in the response to cellular oxidative stress. Moreover, we found alterations at mRNA level in the mechanisms of telomeric DNA repair. Specifically, increased *RAD51D* mRNA levels were correlated with left ventricular diameters. RAD51D protein levels were unaltered, however, and were inversely corelated with the miR-103a-3p upregulation. We also observed the overexpression of lncRNAs (*TERRA* and *GUARDIN)* involved in telomere protection in response to stress and alterations in their regulatory molecules. Expression of the *TERRA* transcription factor *ATF7* was correlated with superoxide dismutase 1 expression and left ventricular diameters. The levels of *GUARDIN* and its transcription factor *FOSL2* were correlated with those of catalase. Therefore, we showed specific alterations in the mechanisms of telomeric DNA repair and protection, and these alterations are related to an increase in the response mechanisms to oxidative stress and cardiac dysfunction in ICM.

## 1. Introduction

Ischaemic cardiomyopathy (ICM) is an aetiologic cause of heart failure (HF) with a high prevalence, and it is characterized by increased oxidative stress, loss of cardiomyocytes, scarring of myocardial tissue, and ventricular failure. The prevalence of ICM is increasing and carries a high mortality rate, with no effective treatment currently available [1].

Different studies suggest the existence of a relationship between telomeric alterations and the development of HF [2,3]. Telomeres are heterochromatic structures that are located at the terminal ends of the chromosomes of eukaryotic cells. In mammals, telomeres consist of tandem repeats of a guanine-rich DNA sequence (TTAGGG) and shelterin complex. This complex is composed of six polypeptides and assembles through the binding of the double stranded TTAGGG repeat binding proteins TRF1 and TRF2 which in turn recruit RAP1, TIN2, TPP1 and POT1 [4]. Recently, it has been described that the telomeric sequence is transcribed by RNA polymerase II, giving rise to a class of long noncoding RNAs (lncRNAs) containing telomeric repeats called *TERRA*. These lncRNAs are involved in the maintenance of telomere homeostasis [5]. Due to limitations of the conventional DNA replication machinery in the absence of maintenance mechanisms, telomeres progressively shorten during subsequent cell divisions. When cells reach a critical telomeric length, telomere protection is lost, and DNA damage response pathways are activated, leading cells, to replicative senescence and/or apoptosis [6,7]. Telomere length has been studied in different cells, such as monocytes, and even cardiac tissue from patients with HF [8,9]. The mechanism by which eukaryotic must counteract this shortening occurs through telomerase, an enzyme that allows the number of telomeric sequences to be expanded. However, this hypothesis is insufficient to explain how senescence affects cells with a low proliferation rate and reduced telomerase levels, such as cardiomyocytes. In addition, cellular senescence may occur independent of telomere shortening, for example, due to DNA damage and oxidative injury [10]. For this reason, other critical processes related to maintenance of the telomere structure are proposed as possible causes of cell death in HF. Among the main proposed mechanisms, oxidative stress was identified [11,12]. Oxidative stress can cause telomere DNA damage, leading to cardiac dysfunction [13]. On the other hand, recent evidence has indicated the roles of ncRNAs, lncRNAs and miRNAs, in telomere biology regulation and their involvement in telomere dysfunction and cell senescence [14], such as *GUARDIN* lncRNA, although the role of these molecules in the progression of HF is unknown. 

Therefore, elucidating the molecular mechanisms responsible for maintaining telomeres in heart tissue would provide valuable information about cardiac function. In the present study, we examined changes at the RNA level in protein-coding genes and non-protein coding genes involved in telomere homeostasis and the oxidative stress response, as well as the relationship that exists between both processes in the cardiac tissues of patients with ICM compared to control subjects (CNT). Moreover, we analysed the protein levels of key molecules in the maintenance of telomeres. Additionally, we evaluated the relationship between altered gene expression and left ventricular (LV) dysfunction.

## 2. Materials and Methods

### 2.1. Tissue Sample Collection

Left ventricle samples (≈85% cardiomyocytes [15]) obtained from explanted human hearts were used in our experiments. Tissue samples were obtained from the region near the LV apex of each explanted heart. After extraction, they were kept in 0.9% NaCl at 4 °C for a maximum of 6 h after loss of coronary circulation. Samples were stored at −80 °C until further analysis.

All available data were collected for each patient: clinical history, electrocardiograms, Doppler echocardiography, hemodynamic studies, and coronary angiography. Patients with primary valve disease were excluded from the study. Patients were classified according to the functional criteria of the New York Heart Association and were receiving medical treatment according to the guidelines of the European Society of Cardiology.

CNT samples were obtained from hearts of non-diseased donors who had been rejected for heart transplantation due to size or blood group incompatibility and the inability to find a new recipient during the transplant window. For these donors, the cause of death was stroke or car accidents. All CNT hearts showed normal LV function (ejection fraction (EF) > 50%), as determined by Doppler echocardiography, and had no history of heart disease. Only age and gender data were available, in accordance with the Spanish Organic Law on Data Protection 15/1999.

This study was approved by the Ethics Committee (Biomedical Investigation Ethics Committee of La Fe University Hospital, Spain). The investigation conforms to the principles outlined in the Declaration of Helsinki [16] and all tissue samples were obtained with the written informed consent of the patients or their close relatives. 

### 2.2. RNA Extraction and Quality Assessment

TRIzol^®^ agent was used to homogenize tissue samples in TissueLyser LT (Qiagen; Manchester, UK). RNA was extracted using the PureLink ™ Kit (Ambion Life Technologies; Waltham, MA, USA) for mRNA sequencing (mRNA-seq) and the Quik-RNA^TM^ miniprep plus kit (Zymo Research; Irvine, CA, USA) for ncRNA sequencing (ncRNA-seq), in both cases following the manufacturer’s recommendations. The RNA concentration was measured on the Nanodrop 1000 spectrophotometer (Thermo Fisher Scientific; Horsham, UK), and the purity and integrity of RNA samples were measured using the microfluidics-based platform 2100 Bioanalyzer with the RNA 6000 Nano LabChip Kit (Agilent Technologies; Spain). All RNA samples displayed at 260/280 absorbance ratio ≥2.0 and reached a minimal ≥ RIN of 9.

### 2.3. mRNA Sequencing

For mRNA-seq 23 samples were analysed (ICM, *n* = 13; and CNT, *n* = 10) through SOLiD 5500XL platform. Methods used for sequencing, computational analysis, and gene functional annotation of the mRNA-seq data were performed as previously described by Roselló-Lletí et al. [17]. The data presented in this manuscript have been deposited in the NCBI’s Gene Expression Omnibus (GEO) database and are accessible through the GEO series accession number GSE55296.

### 2.4. Gene Functional Enrichment

We performed a functional enrichment analysis of differentially expressed genes based on hypergeometric testing using the ToppGene suite [18]. We selected the differentially expressed genes from ICM patients with *p* value < 0.05 by using the FDR correction.

### 2.5. ncRNA Sequencing

For this analysis, 30 samples were used (ICM, *n* = 22; and CNT, *n* = 8). The cDNA libraries have been obtained following Illumina’s recommendations. Briefly, 3´ and 5´adaptors were sequentially ligated to the RNA prior to reverse transcription and cDNA generation. The cDNA was enriched using PCR to create an indexed double-stranded cDNA library, and size selection (20–150 nucleotides) was performed using a 6% polyacrylamide gel. The quality and quantity of the libraries were analysed using a 4200 TapeStation D1000 High-Sensitivity assay. The cDNA libraries were pooled, and the pools were sequenced using paired-end sequencing (100 × 2) in the Illumina HiSeq 2500 sequencer. 

Quality control of the raw sequence data was performed using FastQC software. Bias was prevented through adapter identification and elimination using Trim Galore [19]. For the possible *TERRA* counts reads, the profile (TTAGGG)×4 was searched using FIMO algorithm [20]. A threshold of adjusted *p* value by FDR of 0.05 was used for positive reads.

### 2.6. Western Blot

For Western blot 44 samples were analysed (ICM, *n* = 34; and CNT, *n* = 10). Methods used for homogenization of samples, protein determination, polyacrylamide gel electrophoresis and Western blot analysis were performed as previously described by Roselló-Lletí et al. [17]. Specifically, we use Bis-Tris electrophoresis on 4–12% polyacrylamide gels under reducing conditions. The primary detection antibodies used were anti-Tin2 rabbit monoclonal antibody (1:500), anti-RAP1 mouse monoclonal antibody (1:500), anti-Tankyrase rabbit polyclonal antibody (1:500), anti-RAD51D rabbit monoclonal antibody (1:500), and anti-GAPDH mouse monoclonal antibody (1:500) as a loading control, all of them obtained from Abcam.

### 2.7. Statistical Analysis

Data were expressed as the mean ± standard deviation (SD) for continuous variables and as percentage values for discrete variables. The Kolmogorov–Smirnov test was applied for analysing the data distribution. Significant mean differences between groups with a normal distribution were analysed using the Student’s *t*-test, whereas the non-parametric Mann–Whitney U test was performed for comparisons between data that were non-normally distributed. Clinical characteristics of patients were compared using Student’s *t*-test for continuous variables and Fisher’s exact test for discrete variables. Pearson’s correlation coefficient was calculated to analyse the association between normal variables. Significance was defined for *p* values < 0.05. All statistical analyses were performed using the SPSS software (version 20.0) for Windows (IBM SPSS Inc., Chicago, IL, USA).

## 3. Results

### 3.1. Clinical Characteristics of Patients

As shown in Table 1, all ischemic patient populations included in the different studies were homogeneous according to the clinical characteristics of patients. All patients were men (98%) and their mean age was 55 ± 7 years. All patients presented an NYHA functional classification between III-IV and had previously been diagnosed with significant comorbidities, including hypertension (33–52%) and diabetes mellitus (42–52%). The CNT group consisted mainly of men (65%) with a mean age of 55 ± 17 years. Comorbidities and other echocardiographic data were not available for the CNT group, in accordance with the Spanish Organic Law on Data Protection 15/1999.

### 3.2. Telomere Homeostasis Alterations in ICM Patients. Relationship with Cardiac Function Parameters

To investigate the changes in mRNA expression of molecules involved in telomere homeostasis between patients with ICM and CNT individuals, we performed a large-scale gene expression screen using RNA-seq technology, with a SOLiD 5500XL sequencer. First to all, after differential expression genes were obtained, GO enrichment analysis was performed to classify differentially expressed genes according to their functions, and to annotate and classify these genes. Significant GO annotations of molecular functions, biological process and cellular components are represented in the Figure S1.

**Figure 1:**
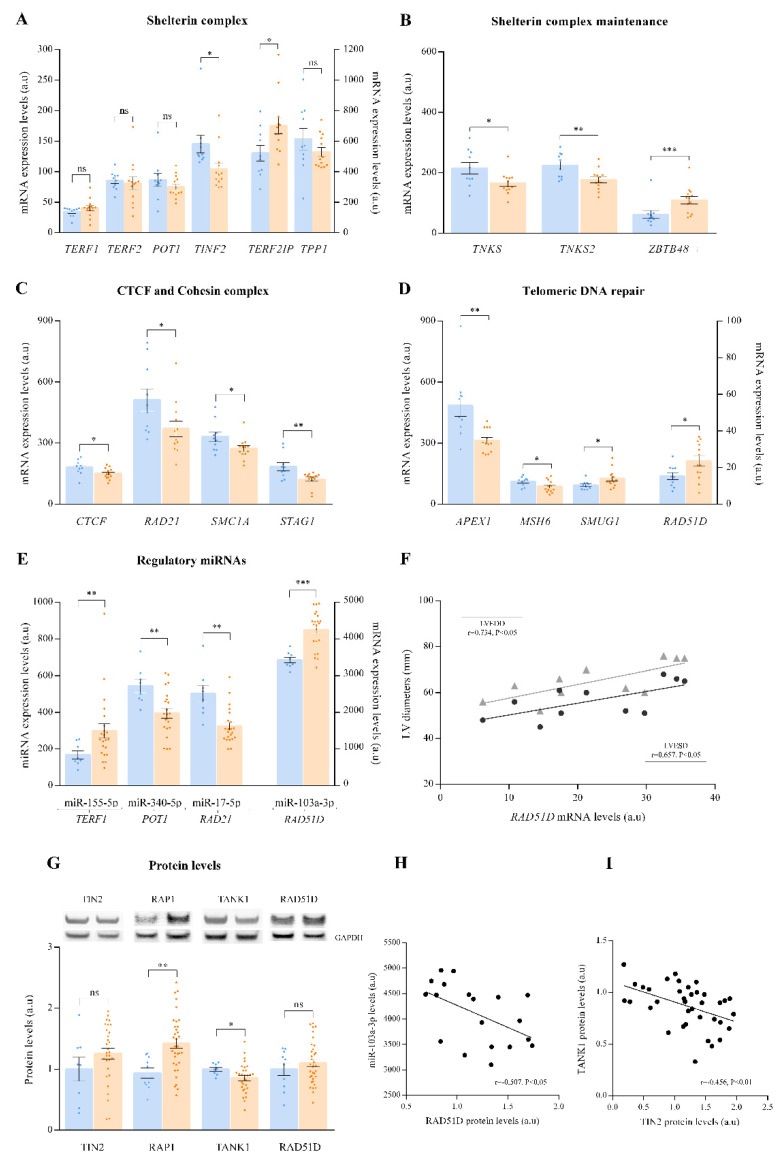
Dot plot graph of mRNA, miRNA and protein expression levels of main molecules associated with telomere homeostasis. (**A**) Genes of shelterin complex. (**B**) Genes involved in the maintenance shelterin complex. (**C**) *CTCF* and cohesin complex genes. (**D**) Genes involved in the telomere DNA repair. (**E**) miRNAs targeting *TERF1*, *POT1*, *RAD21* and *RAD51D*. (**F**) Correlation of *RAD51D* mRNA levels with left ventricular end-systolic (LVESD) and left ventricular end-diastolic (LVEDD) diameters. (**G**) TIN2, RAP1, TANK1 and RAD51D protein levels. (**H**) Correlation of RAD51D protein levels with miR-103a-3p expression levels. (**I**) Correlation of TIN2 with TANK1 protein levels. The results were obtained by mRNA-sequencing SOLiD 5500XL platform and ncRNA-sequencing Illumina HiSeq 2500 platform. Data are presented as the mean ± SEM. au, arbitrary units. Ischemic cardiomyopathy patients (orange), controls subjects (blue). Left ventricular end-systolic diameter (black), left ventricular end-systolic diameter (grey). * *p* < 0.05, ** *p* < 0.01, *** *p* < 0.0001.

We focused on the analysis of molecules related to structural elements of the telomere (shelterin and cohesin complex) and telomeric DNA repair (Table S1). The expression levels of the constituent molecules of the shelterin complex, *TERF1*, *TERF2*, *POT1*, *TINF2*, *TERF2IP* and *TPP1*, were analysed (Figure 1A). We observed alterations in two components, *TINF2* (FC = −1.39, *p* < 0.05) and *TERF2IP* (FC = 1.34, *p* < 0.05), as well as the underexpression of genes involved in the correct assembly of the shelterin complex, *TNKS* (FC = −1.30, *p* < 0.05) and *TNKS2* (FC = −1.27, *p* < 0.01), and overexpression of *ZBTB48* (FC = 2.20, *p* < 0.0001), a molecule that competes with the binding of the shelterin complex to the telomere (Figure 1B). In addition, *CTCF* (FC = −1.21, *p* < 0.05) and several subunits of the cohesin complex (*RAD21* (FC = −1.38, *p* < 0.05), *SMC1A* (FC = −1.21, *p* < 0.05), and *STAG1* (FC = −1.50, *p* < 0.01)), key components in telomere protection, were underexpressed in ICM (Figure 1C). On the other hand, we determined that telomeric DNA repair mechanisms were altered in ICM patients; specifically, we observed dysregulation in the expression of genes associated with telomeric stability (Figure 1D), such as *APEX1* (FC = −1.55, *p* < 0.01), *MSH6* (FC = −1.29, *p* < 0.05), *SMUG1* (FC = 1.36, *p* < 0.05) and *RAD51D* (FC = 1.53, *p* < 0.05).

The expression levels of the described miRNAs regulating the molecules of telomere homeostasis were analysed (Table S1). We observed overexpression of miR-155-5p (FC = 1.77, *p* < 0.01) and miR-103a-3p (FC = 1.24, *p* < 0.0001), whose targets are *TERF1* and *RAD51D* respectively, as well as underexpression of miR-340-5p (FC = −1.27, *p* < 0.01) and miR-17-5p (FC = −1.36, *p* < 0.01), whose targets are *POT1* and *RAD21*, respectively (Figure 1E).

Furthermore, we observed significant correlations between one of the main genes involved in telomeric DNA repair, *RAD51D*, and cardiac function parameters (Figure 1F). Echocardiographic data were available in eleven of the thirteen individuals. *RAD51D* mRNA levels were positively correlated with both LV end-systolic (r = 0.657, *p* < 0.05) and end-diastolic diameters (r = 0.734, *p* < 0.05).

On the other hand, we performed Western blots of several molecules related to telomere homeostasis (Figure 1G). Specifically, we focused on the study of the shelterin complex molecule TIN2, which is encoded by the *TINF2* gene. TIN2 showed similar protein levels between ICM patients and controls. In addition, we also analyzed RAP1 protein, enconded by *TERF2IP*. In concordance with the upregulation of *TERF2IP,* our results reveal an increase in RAP1 protein levels (FC = 1.42, *p* < 0.01). Regarding the maintenance of the shelterin complex, TANK1 showed lower protein levels in the ischemic group (FC= −1.17, *p* < 0.05), in the same way as the gene that encodes it, *TNKS*. Moreover, we analysed the protein levels of the molecule responsible for telomeric DNA repair, *RAD51D,* and observed similar RAD51D protein levels between controls and ischemic patients. Interestingly, patients shared in both assays showed inverse correlation between RAD51D protein levels and the overexpression of miR-103a-3p (r = −0.507, *p* < 0.05; Figure 1H). Furthermore, TIN2 and TANK1 protein expression showed an inverse correlation (r = −0.456, *p* < 0.01, Figure 1H).

### 3.3. TERRA and GUARDIN Regulation. Relationship with Cardiac Function Parameters

**Figure 2:**
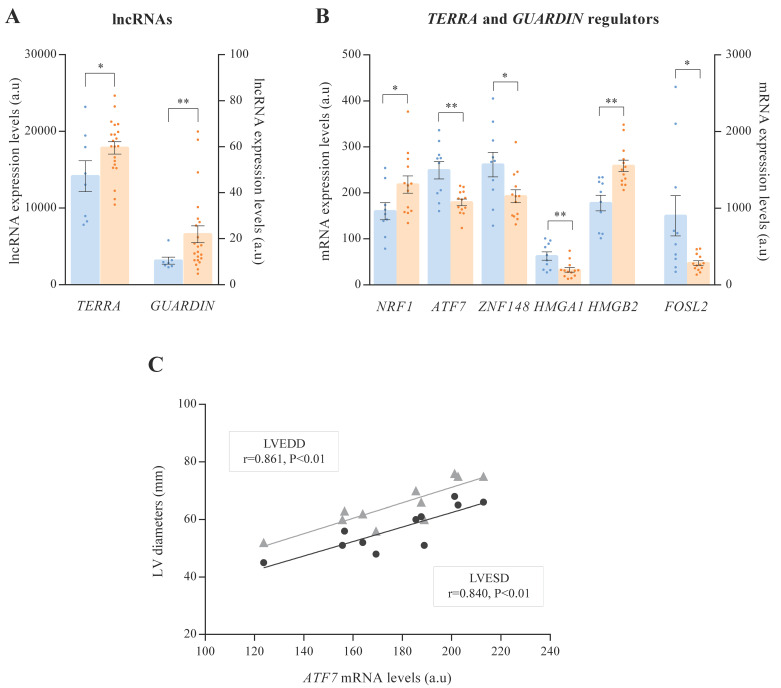
Dot plot graph of lncRNAs expression and mRNA expression levels of altered genes associated with *TERRA* and *GUARDIN* regulation. (**A**) *TERRA* and *GUARDIN* expression. (**B**) Genes involved in *TERRA* and *GUARDIN* regulation. (**C**) Correlation of *ATF7* mRNA levels with left ventricular end-systolic (LVESD) and left ventricular end-diastolic (LVEDD) diameters. The results were obtained by mRNA-sequencing SOLiD 5500XL platform and ncRNA-sequencing Illumina HiSeq 2500 platform. Data are presented as the mean ± SEM. au, arbitrary units. Ischemic cardiomyopathy patients (orange), controls subjects (blue). Left ventricular end-systolic diameter (black), left ventricular end-systolic diameter (grey). * *p* < 0.05, ** *p* < 0.01.

Through ncRNA-seq technology, we estimated the expression of *TERRA* and *GUARDIN*, lncRNAs related to telomeric integrity (Figure 2A). Both lncRNAs were overexpressed in ICM patients (*TERRA*, FC = 1.26, *p* < 0.05; and *GUARDIN*, FC = 2.08, *p* < 0.01). Moreover, we analysed the expression of several molecules involved in the regulation of *TERRA* and *GUARDIN* (Table S2; Figure 2B). We observed alterations in the main transcription factors associated with the *TERRA* promoter [21,22,23]. Specifically, *NRF1* (FC = 1.36, *p* < 0.05) was increased, and *ATF7* (FC = −1.39, *p* < 0.01) and *ZNF148* (FC = −1.36, *p* < 0.05) were decreased in ICM patients. In addition, other molecules related to *TERRA* regulation were altered in ICM [24], such as *HMGA1* (FC = −2.13, *p* < 0.01) and *HMGB2* (FC = 1.46, *p* < 0.01). We also observed a close relationship at the mRNA level between the different genes involved in the regulation of *TERRA* (Table S3). Furthermore, *FOSL2*, a transcription factor regulator of *GUARDIN* expression [25], was underexpressed (FC = −1.68, *p* < 0.05) in ICM patients.

Interestingly, we observed significant correlations between TERRA transcription factors, *ATF7*, and cardiac function parameters. Echocardiographic data were available in eleven of the thirteen individuals. *ATF7* (Figure 2C) was positively correlated with both LV end-systolic (r = 0.840, *p* < 0.01) and end-diastolic diameters (r = 0.861, *p* < 0.01).

### 3.4. Regulation of Oxidative State in ICM and Relationship with Telomere Homeostasis

**Figure 3:**
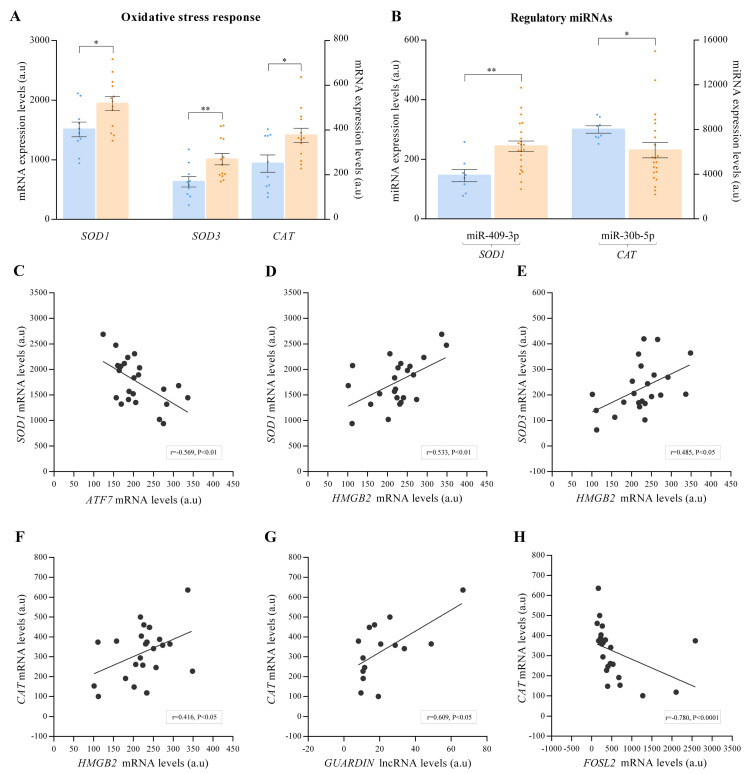
Dot plot graph of mRNA (*n* = 23) and miRNA (*n* = 30) expression levels of altered molecules associated with stress oxidative response. (**A**) mRNA relative expression levels of altered genes associated with stress oxidative response. (**B**) miRNAs target *SOD1* and *CAT*. (**C**) Correlation of *ATF7* mRNA levels with *SOD1* mRNA levels. (**D**) Correlation of *HMGB2* mRNA levels with *SOD1* mRNA levels. (**E**) Correlation of *HMGB2* mRNA levels with *SOD3* mRNA levels. (**F**) Correlation of *HMGB2* mRNA levels with *CAT* mRNA levels. (**G**) Correlation of *GUARDIN* mRNA levels with *CAT* mRNA levels. (**H**) Correlation of *FOSL2* mRNA levels with *CAT* mRNA levels. The results were obtained by mRNA-sequencing SOLiD 5500XL platform and ncRNA-sequencing Illumina HiSeq 2500 platform. Data are presented as the mean ± SEM. au, arbitrary units. Ischemic cardiomyopathy patients (orange), controls subjects (blue). * *p* < 0.05, ** *p* < 0.01.

We analysed changes at the RNA level in key molecules in response to oxidative stress in the cell. We observed the overexpression of superoxide dismutases, *SOD1* (FC = 1.29, *p* < 0.05) and *SOD3* (FC = 1.60, *p* < 0.01), and catalase *CAT* (FC = 1.51, *p* < 0.05) in ICM patients (Figure 3A). Furthermore, the expression levels of the described miRNAs that regulate molecules of the oxidative stress response were analysed. We observed an overexpression of miR-409b-3p (FC = 1.68, *p* < 0.01), a regulator of the expression of *SOD1*, and an underexpression of miR-30b-5p (FC = −1.23, *p* < 0.05), whose target is *CAT* (Figure 3B).

Next, we investigated whether there was any relationship between the altered genes that play a role in the processes of telomere homeostasis and oxidative stress response mechanisms in ICM patients. Important significant relationships were obtained, highlighting those observed in components related to lncRNAs, *TERRA,* and *GUARDIN*. In relation to the TERRA system, we observed that the *ATF7* transcription factor was negatively correlated with *SOD1* (Figure 3C), and the *TERRA* regulatory factor *HMGB2* was positively correlated with the main altered oxidative stress response molecules *SOD1* (Figure 3D), *SOD3* (Figure 3E) and *CAT* (Figure 3F). On the other hand, the mRNA levels of *GUARDIN* (Figure 3G) and its transcription factor *FOSL2* (Figure 3H) were correlated with *CAT*. Furthermore, oxidative stress response genes were correlated with different genes implicated in the maintenance shelterin complex, telomere DNA repair and cohesin complex, which are summarized in Table 2.

## 4. Discussion

Telomeres are dynamic structures whose alterations have been related to pathological states in the cell [26]. The main causes of telomere alterations are decreased telomere-protective factors and increased telomeric risk factors. Specifically, dysregulation of structural elements of the telomere, such as the shelterin complex, has been related to telomere alterations [11]. On the other hand, increases in ROS in the cells are directly related to persistent DNA damage at the telomere regions [13], as well as a close relationship between oxidative stress mechanisms and the state of telomeres has been observed [27,28]. In addition, oxidative stress plays an important role in the pathophysiology of ICM [29]. However, the connections between oxidative stress and telomere biology are complex and incompletely understood in HF [30]. In the present study, our results suggest a deregulation of the main telomere-protective factors, such as alterations in shelterin and cohesin complexes, deregulation of the repair mechanisms of telomeric DNA, and overexpression of *TERRA* and *GUARDIN*, key lncRNAs in telomere protection in response to stress. Moreover, we showed a close relationship between the expression of molecules involved in the maintenance of telomere integrity and the activation of stress response mechanisms, as well as cardiac dysfunction in patients with ICM. 

The shelterin complex plays a main role in the protection of telomeres against external agents and the maintenance of genomic stability in cells [31]. TIN2 is encoded by *TINF2* gene, we observed that at the mRNA levelthis gene was underexpressed in ICM patients. TIN2 is a key component in the correct assembly of the shelterin complex in telomeres, since it allows the union of TRF1 and TRF2 to the heterodimer formed by POT1/TPP1 [32]. We also observed underexpression of the *TNKS* and *TNKS2* genes. Both molecules are required for telomere separation in telomere elongation [33]. Additionally, TIN2 formed a ternary complex with TRF1 and tankyrase 1 and stabilized their interaction regulating telomere length [34], although TIN2 protein levels are not altered in patients with ICM, its expression is related to the expression of Tankyrase, which is decreased in patients with ICM. In addition, we observed overexpression of the *ZBTB48* gene. This molecule competes with the different units of the shelterin complex, preventing its binding to the telomere and favoring telomeric shortening [35]. These results suggest alterations in the correct assembly of the shelterin complex, which may affect its fundamental role in chromosomal protection and the regulation of telomere length in the ICM. On the other hand, Lototska et al. [36] recently described the protective role of RAP1 in telomeric shortening caused by cell stress. In the ICM, we observed that *TERF2IP*, the gene that encodes RAP1, was overexpressed and positively correlated with different superoxide dismutases, which play a fundamental role in the elimination of ROS. In addition, RAP1 protein levels were also upregulated. These results suggest RAP1 can be used as a possiblecompensatory mechanism for the stress present in cardiomyocytes. Previously, it was observed that murine models with Mn-SOD deficiency present cardiac failure, suggesting that oxidative stress could affect the activity of myocardial telomerase and telomere-associated proteins [37].

*CTCF* and cohesin complex have also been described as integral components of most human subtelomeres, which participate in chromatin organization and telomere end protection. Our results showed underexpression of *CTCF* and several components of the cohesin complex, such as *RAD21*, *SMC1A* and *STAG1* in ICM patients. Cohesin subunit SA1, encoded by *STAG1,* is recruited to telomere repeats by the shelterin protein TIN2, and this interaction is required for telomere stability [38]. Both molecules were underexpressed and were able to promote the destabilization of telomeres in ICM. In addition, depletion of either CTCF or RAD21 caused telomere-induced DNA damage foci formation and destabilized TRF1 and TRF2 binding to TTAGGG proximal subtelomere DNA in human cell culture [39].

On the other hand, it has been widely described that patients with ICM present a higher production of ROS that triggers DNA sequence damage, which induces cellular senescence [40]. We observed alterations in the expression of several genes involved in the telomere DNA repair pathway. Specifically, the *SMUG1* gene encodes a glycosylase enzyme, which is primarily responsible for removing damaged bases in DNA. It has recently been discovered that this molecule is also necessary for the correct maturation of the enzyme telomerase and has profound implications in telomeric homeostasis [41]. Our results showed that *SMUG1* gene expression is increased in ICM patients. We also found overexpression of the *RAD51D* gene, which is related to telomeric DNA repair [42], and also drives reversed replication fork formation and mediates active fork slowing upon mild genotoxic stress [43,44]. In addition, miR-103a-3p, was increased in ICM patients. miR-103a-3p functions by regulating the expression of RAD51D protein [45]. In this sense, we did not observe differences in the level of RAD51D protein in patients with ICM compared to controls. Furthermore, we observed a significant correlation between *RAD51D* gene expression and ventricular function parameters, but the role of this pathway in the pathophysiology of the disease is unknown and represents an interesting area of research. 

In recent years many investigations have been directed to the study of the role of ncRNAs in the DNA damage response, the protection of telomere ends, and the maintenance of telomeres. Several miRNAs have been described as regulators of the expression of factors that influence telomere dynamics [14]. We observed alterations in the expression of miRNAs previously described, such as regulators of the molecules involved in telomere homeostasis and oxidative stress response [46,47,48]. Specifically, we observed an increase in miR-155-5p in ICM patients, whose expression is associated with telomere and genomic instability. In breast cancer cells, the increase in miR-155 represses translation of the TRF1 protein, preventing the correct assembly of the shelterin complex at the telomeres [49]. We also observed underexpression of miR-30b-5p together with overexpression of catalase mRNA. Haque et al. [50] described that increased miR-30b inhibits endogenous catalase expression in human cells. In addition, in acute myocardial ischaemic patients a reduction in miR-30b levels in serum has been described [51]. LncRNAs are key molecules in telomere integrity, highlighting the role of *TERRA* and *GUARDIN*. The lncRNA *GUARDIN,* a transcriptional target of TP53, was overexpressed. Increased *GUARDIN* maintains genome integrity in the presence of DNA damage [52]. Sun et al. [25] described that FOSL2 acts as a transcriptional repressor of *GUARDIN,* and the inhibition of *FOSL2* by rapamycin promotes an increase in *GUARDIN* that activates the p21-dependent pathway reducing cellular senescence. In the present study, we observed a reduction in *FOSL2* levels together with an increase in *GUARDIN*, as well as a correlation between the expression of both molecules and catalase levels, which could act as a response mechanism to the increase in cell damage in ICM patients. In addition, *GUARDIN* protects telomere ends from damage in large part by sequestering miR-23a, thereby ensuring the production of the shelterin component TRF2 [52]. However, we did not observe changes in the expression of miR-23a and *TERF2,* and there may be regulation at the posttranscriptional level. The lncRNA *TERRA*, the main transcript of the telomeric sequence, is involved in the regulation of telomerase, formation of heterochromatin at telomeres and proper capping of chromosome ends [5]. Our results suggested overexpression of *TERRA*. In the same way, a preliminary study has been recently published describing an increase in *TERRA* in ICM patients [53]. In other diseases characterized by an increase in ROS, overexpression of *TERRA* has also been described. This increase has been associated with telomere dysfunctions caused by oxidative stress [54]. In addition, it has been observed that the use of antioxidant treatments reduces the expression of *TERRA* [55]. One possible explanation for this observation is related to the nature of the *TERRA* sequence. Its sequence (5´-UUAGGG-3´) presents a high degree of guanine residues, such as telomeres, and these molecules are highly prone to oxidation. Therefore, the binding of *TERRA* to telomeres plays a protective role against oxidation [56].

On the other hand, the expression of *TERRA* is highly regulated in the cells. At the transcription level, several transcription factors related to the expression of *TERRA* have been described. CTCF and the cohesin complex are also positive regulators of *TERRA* transcription through RNA polymerase II recruitment to the *TERRA* promoter region [39], but the expression of these molecules is reduced in ICM. However, we observed that *NRF1* was overexpressed in ICM patients. NRF1 binds to the *TERRA* promoter, promoting its transcription, in addition to acting as an antioxidant factor [23]. Diman et al. [57] described that intense exercise promotes the production of ROS in human skeletal muscle cells, triggering an increase in the expression of NRF1 and consequently greater expression of *TERRA*. Both molecules could have a protective role against increased stress in cardiac cells. Negative regulators of the expression of *TERRA* were also altered. We observed a reduction in expression of the *ZNF148* and *ATF7* genes in ICM patients, and these genes showed a significant correlation between their expression and ventricular function parameters. Furthermore, transcriptomic analysis in swine has described underexpression of the *ATF7* gene, which is related to LV remodeling after myocardial infarction [58]. The expression of *ATF7* was also correlated with the expression of *SOD1*. *ATF7* is a stress-responsive chromatin regulator, and increased stress on cells causes the release of ATF7 from the *TERRA* gene promoter and the induction of *TERRA* gene transcription [22]. Additionally, different molecules regulating the function of *TERRA* have been described. We observed underexpression of *HMGA1* and overexpression of the *HMGB2* gene. The decrease in *HMGA1* has been related to an increase in the association of *TERRA* with telomeres, while the decrease in *HMGB2* has been related to the reduction of *TERRA* levels without affecting its association with the telomere [24]. In addition, *HMGB2* was correlated with the expression of molecules to response to oxidative stress. Overall, the different molecules related to the regulation of the expression and function of *TERRA* showed close relationships between them, suggesting a high regulation of *TERRA* in ICM patients. Research on *TERRA* regulation in human ICM represents a promising possibility to identify novel pathophysiological mechanisms, and this approach could be a critical step towards more effective personalized care based on potential therapeutic strategies to block ICM progression.

A common limitation of studies examining the cardiac tissues of patients with end-stage HF is the great variability in treatment. However, our study population was aetiologically homogeneous, and all patients were treated according to established clinical guidelines. In addition, it is crucial to emphasize the importance of having carried out this study in a significant number of ICM samples from explanted human hearts undergoing cardiac transplantation and CNT donors, making our results applicable for ICM population. On the other hand, the ncRNA-seq technique does not allow stratification of TERRA reads derived from the telomere regions with respect to another location within the whole human genome. There is no perfect assay capable of measuring the expression of TERRA [59] but ncRNA-seq could be an interesting approximation of the differences regardless of TERRA origin.

## 5. Conclusions

We found relevant alterations in the mRNA level in the shelterin complex and telomeric DNA repair, highlighting the changes in expression levels of the *RAD51D* gene and their relationship with cardiac function parameters. We demonstrated that overexpression of miR-103a-3p is inversely related to the protein levels of its target, RAD51D. We showed that the lncRNAs *TERRA,* and *GUARDIN,* and their regulatory molecules such as *ATF7*, *HMGB2,* and *FOSL2,* could play relevant roles in telomere protection in response to oxidative stress in the ICM.

## Figures and Tables

**Table 1:** Clinical characteristics of ischemic cardiomyopathy patients.

	mRNA-seq	ncRNA-seq	Western Blot
	ICM (*n* = 13)	ICM (*n* = 18)	ICM (*n* = 34)
Age (years)	54 ± 8	55 ± 8	54 ± 7
Gender male (%)	100	100	97
*NYHA* class	III-IV	III-IV	III-IV
BMI (kg/m^2^)	27 ± 4	26 ± 3	27 ± 4
Haemoglobin (mg/dL)	14 ± 3	14 ± 2	13 ± 2
Haematocrit (%)	41 ± 6	41 ± 4	40 ± 6
Total cholesterol (mg/dL)	162 ± 41	175 ± 46	178 ± 45
Prior hypertension (%)	33	35	52
Prior smoking (%)	92	78	87
Diabetes mellitus (%)	42	47	52
LVEF (%)	24 ± 4	23 ± 6	23 ± 7
LVESD (mm)	56 ± 8	53 ± 8	55 ± 8
LVEDD (mm)	64 ± 8	62 ± 9	63 ± 8

ICM, ischemic cardiomyopathy; NYHA, New York Heart Association; BMI, body mass index; LVEF, ejection fraction; LVESD, left ventricular end-systolic diameter; LVEDD, left ventricular end-diastolic diameter.

**Table 2:** Relationships between altered telomere homeostasis and oxidative stress genes expressed differentially in patients with ischemic cardiomyopathy.

Oxidative Stress Genes	Telomere Homeostasis Genes	Main Function	r	*p* Value
*CAT*	*APEX1*	Telomeric DNA repair	−0.555	<0.01
	*SMC1A*	Cohesin complex	−0.678	<0.0001
*SOD1*	*TERF2IP*	Shelterin complex	0.548	<0.01
	*TNKS*	Shelterin complex maintenance	−0.419	<0.05
*SOD3*	*TERF2IP*	Shelterin complex	0.520	<0.05
	*TNKS*	Shelterin complex maintenance	−0.466	<0.05
	*ZBTB48*	Shelterin complex maintenance	0.673	<0.01
	*APEX1*	Telomeric DNA repair	−0.540	<0.01
	*SMUG1*	Telomeric DNA repair	0.596	<0.01
	*STAG1*	Cohesin complex	−0.548	<0.01

## Data Availability

The mRNA-seq data discussed in this publication have been deposited in NCBI’s Gene Expression Omnibus and are accessible through GEO Series accession number GSE55296 (http://www.ncbi.nlm.nih.gov/geo/query/acc.cgi?acc=GSE55296, accessed on 28 April 2014).

## Supplementary Materials

The following are available online at https://www.mdpi.com/article/10.3390/antiox10111750/s1, Figure S1: Dot plot showing Gene Ontology analyses performed for differentially expressed genes (*p* < 0.05) between ischemic cardiomyopathy patients and controls. Table S1: Molecules involved in telomere homeostasis in ischemic cardiomyopathy. Table S2: Molecules involved in *TERRA* and *GUARDIN* regulation. Table S3: Relationships between *TERRA* regulation genes expressed differentially in patients with ischemic cardiomyopathy.
