# Supplementary material for: Relationships of Telomere Homeostasis with Oxidative Stress and Cardiac Dysfunction in Human Ischaemic Hearts
Source: Antioxidants (Basel). 2021 Nov 1;10(11):1750. doi: 10.3390/antiox10111750 (PMC8615212; doi:10.3390/antiox10111750)
Supplement: Supplementary file 1 [file antioxidants-10-01750-s001.zip › antioxidants-1388049-supplementary.pdf]

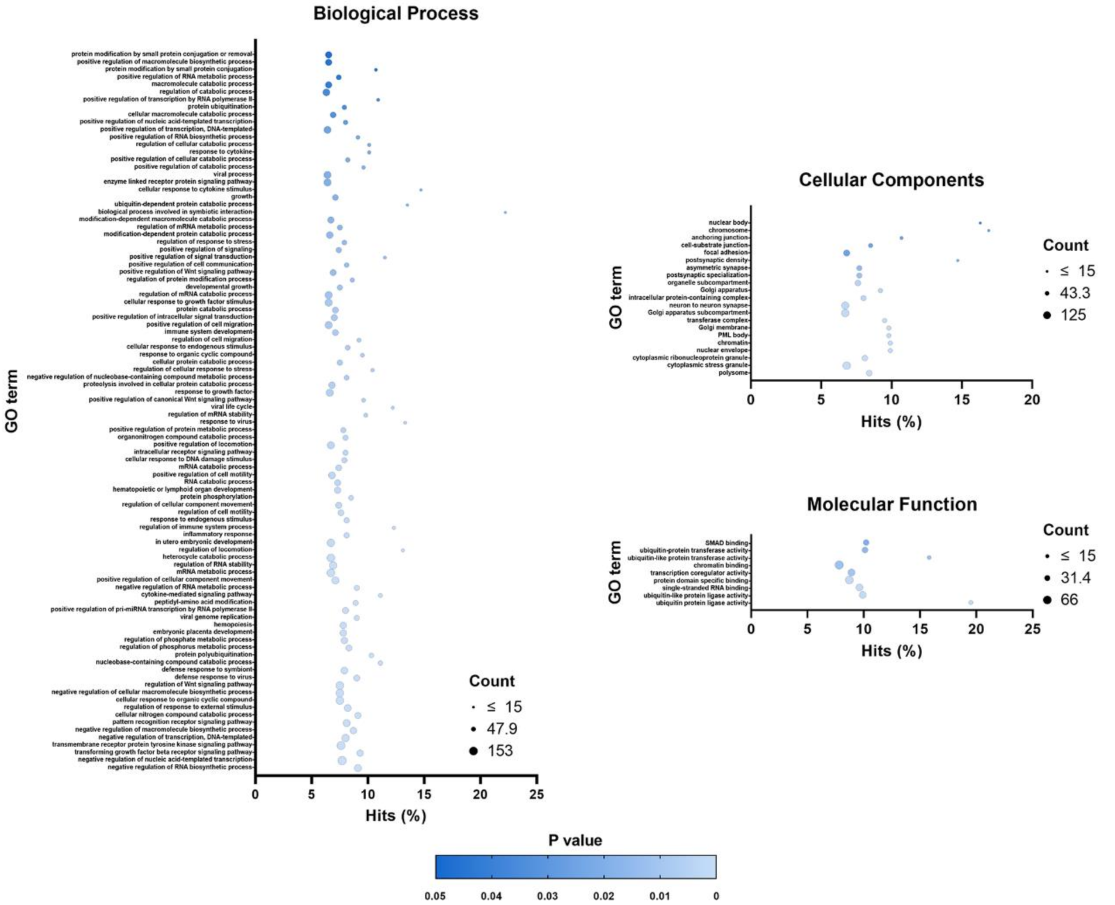


**Figure S1.** Dot plot showing Gene Ontology analyses performed for differentially expressed genes (P < 0.05) between ischemic cardiomyopathy patients and controls.

**Table S1.** Molecules involved in telomere homeostasis in ischemic cardiomyopathy.

**Shelterin complex**

| | ID | Gene | Protein (UniProtKB) | References |
|---|---|---|---|---|
| 1 | ENSG00000128513 | *POT1* | Protection of telomeres 1 | [1] |
| 2 | ENSG00000166848 | ***TERF2IP (RAP1)*** | Telomeric repeat-binding factor 2-interacting protein 1 | [2] |
| 3 | ENSG00000092330 | ***TINF2 (TIN2)*** | TERF1-interacting nuclear factor 2 | [3] |
| 4 | ENSG00000166340 | *TPP1* | ACD shelterin complex subunit and telomerase recruitment factor | [4] |
| 5 | ENSG00000147601 | *TRF1 (TERF1)* | Telomeric repeat-binding factor 1 | [5] |
| 6 | ENSG00000132604 | *TRF2 (TERF2)* | Telomeric repeat-binding factor 2 | [6] |

**Shelterin complex maintenance**

| | ID | Gene | Protein (UniProtKB) | References |
|---|---|---|---|---|
| 7 | ENSG00000139154 | *AEBP2* | Zinc finger protein AEBP2 | [7] |
| 8 | ENSG00000149311 | *ATM* | Serine-protein kinase ATM | [8] |
| 9 | ENSG00000175054 | *ATR* | Serine/threonine-protein kinase ATR | [8] |
| 10 | ENSG00000076108 | *BAZ2A* | Bromodomain adjacent to zinc finger domain protein 2A | [9] |
| 11 | ENSG00000074266 | *EED* | Polycomb protein EED | [10] |
| 12 | ENSG00000108799 | *EZH1* | Histone-lysine N-methyltransferase EZH1 | [10] |
| 13 | ENSG00000106462 | *EZH2* | Histone-lysine N-methyltransferase EZH2 | [10] |
| 14 | ENSG00000127483 | *HP1BP3* | Heterochromatin protein 1-binding protein 3 | [11] |
| 15 | ENSG00000008083 | *JARID2* | Protein Jumonji | [12] |
| 16 | ENSG00000004487 | *KDM1A* | Lysine-specific histone demethylase 1A | [13] |
| 17 | ENSG00000123562 | *MORF4L2* | Mortality factor 4-like protein 2 | [14] |
| 18 | ENSG00000020922 | *MRE11* | Double-strand break repair protein MRE11 | [15] |
| 19 | ENSG00000143033 | *MTF2* | Metal-response element-binding transcription factor 2 | [7] |
| 20 | ENSG00000085840 | *ORC1* | Origin recognition complex subunit 1 | [16] |
| 21 | ENSG00000112511 | *PHF1* | PHD finger protein 1 | [7] |
| 22 | ENSG00000119403 | *PHF19* | PHD finger protein 19 | [7] |
| 23 | ENSG00000132383 | *RPA1* | Replication protein A 70 kDa DNA-binding subunit | [17] |

| | | | | |
|---|---|---|---|---|
| 24 | ENSG00000117748 | *RPA2* | Replication protein A 32 kDa subunit | [18] |
| 25 | ENSG00000106399 | *RPA3* | Replication protein A 14 kDa subunit | [18] |
| 26 | ENSG00000080503 | *SMARCA2* | Probable global transcription activator SNF2L2 | [19] |
| 27 | ENSG00000178691 | *SUZ12* | Polycomb protein SUZ12 | [10] |
| 28 | ENSG00000101945 | *SUV39H1* | Histone-lysine N-methyltransferase SUV39H1 | [20] |
| 29 | ENSG00000173273 | ***TNKS*** | Poly [ADP-ribose] polymerase tankyrase-1 | [21] |
| 30 | ENSG00000107854 | ***TNKS2*** | Poly [ADP-ribose] polymerase tankyrase-2 | [21] |
| 31 | ENSG00000204859 | ***ZBTB48*** | Telomere zinc finger-associated protein | [22] |

**CTCF and Cohesin complex**

| | ID | Gene | Protein (UniProtKB) | References |
|---|---|---|---|---|
| 32 | ENSG00000102974 | ***CTCF*** | Transcriptional repressor CTCF | [23] |
| 33 | ENSG00000164754 | ***RAD21*** | Double-strand-break repair protein rad21 homolog | [23] |
| 34 | ENSG00000072501 | ***SMC1A*** | Structural maintenance of chromosomes protein 1a | [24] |
| 35 | ENSG00000077935 | *SMC1B* | Structural maintenance of chromosomes protein 1B | [25] |
| 36 | ENSG00000108055 | *SMC3* | Structural maintenance of chromosomes protein 3 | [26] |
| 37 | ENSG00000118007 | ***STAG1*** | Cohesin subunit SA-1 | [27] |
| 38 | ENSG00000101972 | *STAG2* | Cohesin subunit SA-2 | [28] |
| 39 | ENSG00000066923 | *STAG3* | Cohesin subunit SA-3 | [29] |
| 40 | ENSG00000174353 | *STAG3L3* | STAG3-like protein 3 | [30] |

**Telomeric DNA repair**

| | ID | Gene | Protein (UniProtKB) | |
|---|---|---|---|---|
| 41 | ENSG00000100823 | ***APEX1*** | DNA-(apurinic or apyrimidinic site) endonuclease | [31] |
| 42 | ENSG00000012061 | *ERCC1* | DNA excision repair protein ERCC-1 | [32] |
| 43 | ENSG00000163161 | *ERCC3* | DNA excision repair protein ERCC-3 | [33] |
| 44 | ENSG00000225830 | *ERCC6* | DNA excision repair protein ERCC-6 | [34] |
| 45 | ENSG00000188486 | *H2AFX* | Histone H2AX | [35] |
| 46 | ENSG00000095002 | *MSH2* | DNA mismatch repair protein Msh2 | [36] |

| | | | | |
|---|---|---|---|---|
| 47 | ENSG00000116062 | ***MSH6*** | DNA mismatch repair protein Msh6 | [37] |
| 48 | ENSG00000065057 | *NTHL1* | Endonuclease III-like protein 1 | [38] |
| 49 | ENSG00000114026 | *OGG1* | N-glycosylase/DNA lyase | [39] |
| 50 | ENSG00000122512 | *PMS2* | Mismatch repair endonuclease PMS2 | [40] |
| 51 | ENSG00000152942 | *RAD17* | Cell cycle checkpoint protein RAD17 | [41] |
| 52 | ENSG00000185379 | ***RAD51D*** | DNA repair protein RAD51 homolog 4 | [42] |
| 53 | ENSG00000123415 | ***SMUG1*** | Single-strand selective monofunctional uracil DNA glycosylase | [43] |
| 54 | ENSG00000076248 | *UNG* | Uracil-DNA glycosylase | [44] |
| 55 | ENSG00000154767 | *XPC* | DNA repair protein complementing XP-C cells | [45] |

**miRNAs involved in telomere homeostasis**

| | **ID** | **miRNA** | **Targets** | **References** |
|---|---|---|---|---|
| 56 | ENSG00000284536 | **miR-17-5p** | *RAD21* | [46] |
| 57 | ENSG00000207980 | miR-23a-3p | *TERF2* | [47] |
| 58 | ENSG00000207808 | miR-27a-5p | *APEX1* | [31] |
| 59 | ENSG00000199035 | **miR-103a-3p** | *RAD51D* | [48] |
| 60 | ENSG00000198997 | miR-107 | *RAD51D* | [48] |
| 61 | ENSG00000283904 | **miR-155-5p** | *TERF1* | [49] |
| 62 | ENSG00000208023 | miR-185-5p | *POT1* | [50] |
| 63 | ENSG00000198995 | **miR-340-5p** | *POT1* | [51] |
| 64 | ENSG00000283203 | miR-1246 | *TERF2IP* | [52] |

Molecules marked in bold are altered in patients with ischemic cardiomyopathy.

**Table S2.** Molecules involved in *TERRA* and *GUARDIN* regulation.

| | ID | Gene | Protein (UniProtKB) | References |
|---|---|---|---|---|
| ***TERRA* transcription factors** | | | | |
| 1 | ENSG00000170653 | ***ATF7*** | Cyclic AMP-dependent transcription factor ATF-7 | [53] |
| 2 | ENSG00000120738 | *EGR1* | Early growth response protein 1 | [54] |
| 3 | ENSG00000106459 | ***NRF1*** | Nuclear respiratory factor 1 | [55] |
| 4 | ENSG00000163848 | ***ZNF148*** | Zinc finger protein 148 | [54] |
| ***TERRA* regulator** | | | | |
| 5 | ENSG00000137309 | ***HMGA1*** | High mobility group protein HMG-I/HMG-Y | [56] |
| 6 | ENSG00000164104 | ***HMGB2*** | High mobility group protein B2 | [56] |
| ***GUARDIN* transcription factor** | | | | |
| 7 | ENSG00000075426 | ***FOSL2*** | Fos-related antigen 2 | [57] |

Molecules marked in bold are altered in patients with ischemic cardiomyopathy.

**Table S3.** Relationships between *TERRA* regulation genes expressed differentially in patients with ischemic cardiomyopathy.

| | ***NRF1*** | ***ZNF148*** | ***ATF7*** | ***HMGA1*** |
|---|---|---|---|---|
| ***ZNF148*** | r=-0.426<br>P<0.05 | | | |
| ***ATF7*** | - | r=0.544<br>P<0.01 | | |
| ***HMGA1*** | r=-0.525<br>P<0.05 | - | r=0.436<br>P<0.05 | |
| ***HMGB2*** | - | r=-0.424<br>P<0.05 | r=-0.563<br>P<0.01 | r=-0.424<br>P<0.05 |

## REFERENCES


1. Glousker, G.; Briod, A.S.; Quadroni, M.; Lingner, J. Human shelterin protein POT1 prevents severe telomere instability induced by homology-directed DNA repair. *EMBO J* **2020**, *39*, e104500.
2. Zhang, X.; Liu, Z.; Liu, X.; Wang, S.; Zhang, Y.; He, X.; Sun, S.; Ma, S.; Shyh-Chang, N.; Liu, F.; et al. Telomere-dependent and telomere-independent roles of RAP1 in regulating human stem cell homeostasis. *Protein Cell* **2019**, *10*, 649-667.
3. Frescas, D.; de Lange, T. TRF2-tethered TIN2 can mediate telomere protection by TPP1/POT1. *Mol Cell Biol* **2014**, *34*, 1349-1362.
4. Boyle, J.M.; Hennick, K.M.; Regalado, S.G.; Vogan, J.M.; Zhang, X.; Collins, K.; Hockemeyer, D. Telomere length set point regulation in human pluripotent stem cells critically depends on the shelterin protein TPP1. *Mol Biol Cell* **2020**, *31*, 2583-2596.
5. Zhou, X.Z.; Perrem, K.; Lu, K.P. Role of Pin2/TRF1 in telomere maintenance and cell cycle control. *J Cell Biochem* **2003**, *89*, 19-37.
6. Markiewicz-Potoczny, M.; Lobanova, A.; Loeb, A.M.; Kirak, O.; Olbrich, T.; Ruiz, S.; Lazzerini Denchi, E. TRF2-mediated telomere protection is dispensable in pluripotent stem cells. *Nature* **2021**, *589*, 110-115.
7. Oliviero, G.; Brien, G.L.; Waston, A.; Streubel, G.; Jerman, E.; Andrews, D.; Doyle, B.; Munawar, N.; Wynne, K.; Crean, J.; et al. Dynamic Protein Interactions of the Polycomb Repressive Complex 2 during Differentiation of Pluripotent Cells. *Mol Cell Proteomics* **2016**, *15*, 3450-3460.
8. Tong, A.S.; Stern, J.L.; Sfeir, A.; Kartawinata, M.; de Lange, T.; Zhu, X.D.; Bryan, T.M. ATM and ATR Signaling Regulate the Recruitment of Human Telomerase to Telomeres. *Cell Rep* **2015**, *13*, 1633-1646.
9. Postepska-Igielska, A.; Krunic, D.; Schmitt, N.; Greulich-Bode, K.M.; Boukamp, P.; Grummt, I. The chromatin remodelling complex NoRC safeguards genome stability by heterochromatin formation at telomeres and centromeres. *EMBO Rep* **2013**, *14*, 704-710.
10. Marion, R.M.; Montero, J.J.; Lopez de Silanes, I.; Grana-Castro, O.; Martinez, P.; Schoeftner, S.; Palacios-Fabrega, J.A.; Blasco, M.A. TERRA regulate the transcriptional landscape of pluripotent cells through TRF1-dependent recruitment of PRC2. *Elife* **2019**, *8*.
11. Shi, G.; Hu, Y.; Zhu, X.; Jiang, Y.; Pang, J.; Wang, C.; Huang, W.; Zhao, Y.; Ma, W.; Liu, D.; et al. A critical role of telomere chromatin compaction in ALT tumor cell growth. *Nucleic Acids Res* **2020**, *48*, 6019-6031.
12. Shields, E.J.; Petracovici, A.F.; Bonasio, R. lncRedibly versatile: biochemical and biological functions of long noncoding RNAs. *Biochem J* **2019**, *476*, 1083-1104.
13. Bottoni, G.; Katarkar, A.; Tassone, B.; Ghosh, S.; Clocchiatti, A.; Goruppi, S.; Bordignon, P.; Jafari, P.; Tordini, F.; Lunardi, T.; et al. CSL controls telomere maintenance and genome stability in human dermal fibroblasts. *Nat Commun* **2019**, *10*, 3884.
14. Cusanelli, E.; Chartrand, P. Telomeric repeat-containing RNA TERRA: a noncoding RNA connecting telomere biology to genome integrity. *Front Genet* **2015**, *6*, 143.
15. Panero, J.; Stella, F.; Schutz, N.; Fantl, D.B.; Slavutsky, I. Differential Expression of Non-Shelterin Genes Associated with High Telomerase Levels and Telomere Shortening in Plasma Cell Disorders. *PLoS One* **2015**, *10*, e0137972.
16. Diotti, R.; Loayza, D. Shelterin complex and associated factors at human telomeres. *Nucleus* **2011**, *2*, 119-135.
17. Kim, H.; Li, F.; He, Q.; Deng, T.; Xu, J.; Jin, F.; Coarfa, C.; Putluri, N.; Liu, D.; Songyang, Z. Systematic analysis of human telomeric dysfunction using inducible telosome/shelterin CRISPR/Cas9 knockout cells. *Cell Discov* **2017**, *3*, 17034.
18. Takai, K.K.; Kibe, T.; Donigian, J.R.; Frescas, D.; de Lange, T. Telomere protection by TPP1/POT1 requires tethering to TIN2. *Mol Cell* **2011**, *44*, 647-659.

19. Wu, S.; Ge, Y.; Li, X.; Yang, Y.; Zhou, H.; Lin, K.; Zhang, Z.; Zhao, Y. BRM-SWI/SNF chromatin remodeling complex enables functional telomeres by promoting co-expression of TRF2 and TRF1. *PLoS Genet* **2020**, *16*, e1008799.
20. Porro, A.; Feuerhahn, S.; Delafontaine, J.; Riethman, H.; Rougemont, J.; Lingner, J. Functional characterization of the TERRA transcriptome at damaged telomeres. *Nat Commun* **2014**, *5*, 5379.
21. Hsiao, S.J.; Smith, S. Tankyrase function at telomeres, spindle poles, and beyond. *Biochimie* **2008**, *90*, 83-92.
22. Jahn, A.; Rane, G.; Paszkowski-Rogacz, M.; Sayols, S.; Bluhm, A.; Han, C.T.; Draskovic, I.; Londono-Vallejo, J.A.; Kumar, A.P.; Buchholz, F.; et al. ZBTB48 is both a vertebrate telomere-binding protein and a transcriptional activator. *EMBO Rep* **2017**, *18*, 929-946.
23. Deng, Z.; Wang, Z.; Stong, N.; Plasschaert, R.; Moczan, A.; Chen, H.S.; Hu, S.; Wikramasinghe, P.; Davuluri, R.V.; Bartolomei, M.S.; et al. A role for CTCF and cohesin in subtelomere chromatin organization, TERRA transcription, and telomere end protection. *EMBO J* **2012**, *31*, 4165-4178.
24. Biswas, U.; Stevense, M.; Jessberger, R. SMC1alpha Substitutes for Many Meiotic Functions of SMC1beta but Cannot Protect Telomeres from Damage. *Curr Biol* **2018**, *28*, 249-261 e244.
25. Mannini, L.; Cucco, F.; Quarantotti, V.; Amato, C.; Tinti, M.; Tana, L.; Frattini, A.; Delia, D.; Krantz, I.D.; Jessberger, R.; et al. SMC1B is present in mammalian somatic cells and interacts with mitotic cohesin proteins. *Sci Rep* **2015**, *5*, 18472.
26. Canudas, S.; Smith, S. Differential regulation of telomere and centromere cohesion by the Scc3 homologues SA1 and SA2, respectively, in human cells. *J Cell Biol* **2009**, *187*, 165-173.
27. Remeseiro, S.; Cuadrado, A.; Carretero, M.; Martinez, P.; Drosopoulos, W.C.; Canamero, M.; Schildkraut, C.L.; Blasco, M.A.; Losada, A. Cohesin-SA1 deficiency drives aneuploidy and tumourigenesis in mice due to impaired replication of telomeres. *EMBO J* **2012**, *31*, 2076-2089.
28. Daniloski, Z.; Smith, S. Loss of Tumor Suppressor STAG2 Promotes Telomere Recombination and Extends the Replicative Lifespan of Normal Human Cells. *Cancer Res* **2017**, *77*, 5530-5542.
29. Winters, T.; McNicoll, F.; Jessberger, R. Meiotic cohesin STAG3 is required for chromosome axis formation and sister chromatid cohesion. *EMBO J* **2014**, *33*, 1256-1270.
30. Pezzi, N.; Prieto, I.; Kremer, L.; Perez Jurado, L.A.; Valero, C.; Del Mazo, J.; Martinez, A.C.; Barbero, J.L. STAG3, a novel gene encoding a protein involved in meiotic chromosome pairing and location of STAG3-related genes flanking the Williams-Beuren syndrome deletion. *FASEB J* **2000**, *14*, 581-592.
31. He, H.; Song, F.; Gao, Q.; Lu, Z.; Yuan, Y.; Li, X.; Chen, L.; Jia, C.; Yang, R.; Yang, J.; et al. The APEX1/miRNA-27a-5p axis plays key roles in progression, metastasis and targeted chemotherapy of gastric cancer. *Int J Pharm* **2021**, *599*, 120446.
32. Gay-Bellile, M.; Romero, P.; Cayre, A.; Veronese, L.; Privat, M.; Singh, S.; Combes, P.; Kwiatkowski, F.; Abrial, C.; Bignon, Y.J.; et al. ERCC1 and telomere status in breast tumours treated with neoadjuvant chemotherapy and their association with patient prognosis. *J Pathol Clin Res* **2016**, *2*, 234-246.
33. Ting, A.P.; Low, G.K.; Gopalakrishnan, K.; Hande, M.P. Telomere attrition and genomic instability in xeroderma pigmentosum type-b deficient fibroblasts under oxidative stress. *J Cell Mol Med* **2010**, *14*, 403-416.
34. Batenburg, N.L.; Mitchell, T.R.; Leach, D.M.; Rainbow, A.J.; Zhu, X.D. Cockayne Syndrome group B protein interacts with TRF2 and regulates telomere length and stability. *Nucleic Acids Res* **2012**, *40*, 9661-9674.
35. Pandita, T.K. ATM function and telomere stability. *Oncogene* **2002**, *21*, 611-618.
36. Campbell, M.R.; Wang, Y.; Andrew, S.E.; Liu, Y. Msh2 deficiency leads to chromosomal abnormalities, centrosome amplification, and telomere capping defect. *Oncogene* **2006**, *25*, 2531-2536.

37. Nersisyan, L.; Hopp, L.; Loeffler-Wirth, H.; Galle, J.; Loeffler, M.; Arakelyan, A.; Binder, H. Telomere Length Maintenance and Its Transcriptional Regulation in Lynch Syndrome and Sporadic Colorectal Carcinoma. *Front Oncol* **2019**, *9*, 1172.
38. Vallabhaneni, H.; O'Callaghan, N.; Sidorova, J.; Liu, Y. Defective repair of oxidative base lesions by the DNA glycosylase Nth1 associates with multiple telomere defects. *PLoS Genet* **2013**, *9*, e1003639.
39. Fouquerel, E.; Barnes, R.P.; Uttam, S.; Watkins, S.C.; Bruchez, M.P.; Opresko, P.L. Targeted and Persistent 8-Oxoguanine Base Damage at Telomeres Promotes Telomere Loss and Crisis. *Mol Cell* **2019**, *75*, 117-130 e116.
40. Siegl-Cachedenier, I.; Munoz, P.; Flores, J.M.; Klatt, P.; Blasco, M.A. Deficient mismatch repair improves organismal fitness and survival of mice with dysfunctional telomeres. *Genes Dev* **2007**, *21*, 2234-2247.
41. Boerckel, J.; Walker, D.; Ahmed, S. The Caenorhabditis elegans Rad17 homolog HPR-17 is required for telomere replication. *Genetics* **2007**, *176*, 703-709.
42. Tarsounas, M.; Munoz, P.; Claas, A.; Smiraldo, P.G.; Pittman, D.L.; Blasco, M.A.; West, S.C. Telomere maintenance requires the RAD51D recombination/repair protein. *Cell* **2004**, *117*, 337-347.
43. Kroustallaki, P.; Lirussi, L.; Carracedo, S.; You, P.; Esbensen, Q.Y.; Gotz, A.; Jobert, L.; Alsoe, L.; Saetrom, P.; Gagos, S.; et al. SMUG1 Promotes Telomere Maintenance through Telomerase RNA Processing. *Cell Rep* **2019**, *28*, 1690-1702 e1610.
44. Cortizas, E.M.; Zahn, A.; Safavi, S.; Reed, J.A.; Vega, F.; Di Noia, J.M.; Verdun, R.E. UNG protects B cells from AID-induced telomere loss. *J Exp Med* **2016**, *213*, 2459-2472.
45. Stout, G.J.; Blasco, M.A. Telomere length and telomerase activity impact the UV sensitivity syndrome xeroderma pigmentosum C. *Cancer Res* **2013**, *73*, 1844-1854.
46. Zhao, J.; Fu, W.; Liao, H.; Dai, L.; Jiang, Z.; Pan, Y.; Huang, H.; Mo, Y.; Li, S.; Yang, G.; et al. The regulatory and predictive functions of miR-17 and miR-92 families on cisplatin resistance of non-small cell lung cancer. *BMC Cancer* **2015**, *15*, 731.
47. Luo, Z.; Feng, X.; Wang, H.; Xu, W.; Zhao, Y.; Ma, W.; Jiang, S.; Liu, D.; Huang, J.; Songyang, Z. Mir-23a induces telomere dysfunction and cellular senescence by inhibiting TRF2 expression. *Aging Cell* **2015**, *14*, 391-399.
48. Huang, J.W.; Wang, Y.; Dhillon, K.K.; Calses, P.; Villegas, E.; Mitchell, P.S.; Tewari, M.; Kemp, C.J.; Taniguchi, T. Systematic screen identifies miRNAs that target RAD51 and RAD51D to enhance chemosensitivity. *Mol Cancer Res* **2013**, *11*, 1564-1573.
49. Dinami, R.; Ercolani, C.; Petti, E.; Piazza, S.; Ciani, Y.; Sestito, R.; Sacconi, A.; Biagioni, F.; le Sage, C.; Agami, R.; et al. miR-155 drives telomere fragility in human breast cancer by targeting TRF1. *Cancer Res* **2014**, *74*, 4145-4156.
50. Li, T.; Luo, Z.; Lin, S.; Li, C.; Dai, S.; Wang, H.; Huang, J.; Ma, W.; Songyang, Z.; Huang, Y. MiR-185 targets POT1 to induce telomere dysfunction and cellular senescence. *Aging (Albany NY)* **2020**, *12*, 14791-14807.
51. Li, X.; Zhang, J.; Yang, Y.; Wu, Q.; Ning, H. MicroRNA-340-5p increases telomere length by targeting telomere protein POT1 to improve Alzheimer's disease in mice. *Cell Biol Int* **2021**, *45*, 1306-1315.
52. Qian, M.; Wang, S.; Guo, X.; Wang, J.; Zhang, Z.; Qiu, W.; Gao, X.; Chen, Z.; Xu, J.; Zhao, R.; et al. Hypoxic glioma-derived exosomes deliver microRNA-1246 to induce M2 macrophage polarization by targeting TERF2IP via the STAT3 and NF-kappaB pathways. *Oncogene* **2020**, *39*, 428-442.
53. Liu, B.; Maekawa, T.; Yoshida, K.; Ly, N.H.; Inoue, K.; Hasegawa, A.; Chatton, B.; Ogura, A.; Ishii, S. Telomere shortening by transgenerational transmission of TNF-alpha-induced TERRA via ATF7. *Nucleic Acids Res* **2019**, *47*, 283-298.
54. Feretzaki, M.; Renck Nunes, P.; Lingner, J. Expression and differential regulation of human TERRA at several chromosome ends. *RNA* **2019**, *25*, 1470-1480.

55. Diman, A.; Boros, J.; Poulain, F.; Rodriguez, J.; Purnelle, M.; Episkopou, H.; Bertrand, L.; Francaux, M.; Deldicque, L.; Decottignies, A. Nuclear respiratory factor 1 and endurance exercise promote human telomere transcription. *Sci Adv* **2016**, *2*, e1600031.
56. Scheibe, M.; Arnoult, N.; Kappei, D.; Buchholz, F.; Decottignies, A.; Butter, F.; Mann, M. Quantitative interaction screen of telomeric repeat-containing RNA reveals novel TERRA regulators. *Genome Res* **2013**, *23*, 2149-2157.
57. Sun, X.; Thorne, R.F.; Zhang, X.D.; He, M.; Li, J.; Feng, S.; Liu, X.; Wu, M. LncRNA GUARDIN suppresses cellular senescence through a LRP130-PGC1alpha-FOXO4-p21-dependent signaling axis. *EMBO Rep* **2020**, *21*, e48796.
